# A comparison of the workload of rural and urban primary care physicians in Germany: analysis of a questionnaire survey

**DOI:** 10.1186/1471-2296-12-112

**Published:** 2011-10-11

**Authors:** Jost Steinhaeuser, Stefanie Joos, Joachim Szecsenyi, Antje Miksch

**Affiliations:** 1 Department of General Practice and Health Services Research, University Hospital Heidelberg, Heidelberg, Germany

**Keywords:** Primary Care, Physician Shortage, Rural Area, Germany

## Abstract

**Background:**

Many western countries are facing an existing or imminent shortage of primary care physicians especially in rural areas. In Germany, working in rural areas is often thought to be associated with more working hours, a higher number of patients and a lower income than working in urban areas. These perceptions might be key reasons for the shortage. The aim of this analysis was to explore if working time, number of treated patients per week or proportion of privately insured patients vary between rural and urban areas in Germany using two different definitions of rurality within a sample of primary care physicians including general practitioners, general internists and paediatricians.

**Methods:**

This is a secondary analysis of pre-collected data raised by a questionnaire that was sent to a representative random sample of 1500 primary care physicians chosen by data of the National Association of Statutory Health Insurance Physicians from all federal states in Germany. We employed two different methods of defining rurality; firstly, level of rurality as rated by physicians themselves (urban area, small town, rural area); secondly, rurality defined according to the Organisation for Economic Co-operation and Development.

**Results:**

This analysis was based upon questionnaire data from 715 physicians. Primary care physicians in single-handed practices in rural areas worked on average four hours more per week than their urban counterparts (p < 0.05). Physicians' gender, the number of patients treated per week and the type of practice (single/group handed) were significantly related to the number of working hours. Neither the proportion of privately insured patients nor the number of patients seen per week differed significantly between rural and urban areas when applying the self-rated classification of rurality.

**Conclusion:**

Overall this analysis identified few differences between urban and rural primary care physician working conditions. To counter future misdistribution of primary care, students should receive practical experience in rural areas to get more practical knowledge on working conditions.

## Background

Many western countries face a shortage of primary care physicians especially in rural areas [1,2]. Therefore it is feared that health care systems will not be able to provide sufficient, close to home care to meet the future needs of a growing elderly society [3].

The most crucial concern is how to get primary care physicians to work where they are most needed [4]. In Germany, the *Competence Centre General Practitioners Baden - Wuerttemberg *was founded in the year 2008, to carry out research on the shortage of primary care physicians, in rural areas in particular [5].

Depending on research questions or approaches, rurality is defined in numerous different ways such as cost or time to travel (e.g. to a school or hospital), social representation or geographical concept [6-8]. The Organisation for Economic Co-operation and Development (OECD) defines rurality simply as areas with population densities below 150 inhabitants per square kilometre [9].

In Germany the total average population density is 230 per square kilometre. Federal states in western German have a density of 264 per square kilometre while in the eastern German federal states the density accounts for 152 per square kilometre [10]. Therefore using the OECD definition, there would be almost no rural areas in former West Germany whereas almost all of former East Germany would be defined as rural.

Through previous research projects we have gained insight into the perceptions and beliefs of GP trainees about working in rural areas [11,12]. Many trainees associate working in rural areas with a higher number of working hours ("*seven days a week, 24 hours a day"*) due to a higher number of patients being seen than in urban practices and believe there is less money to be earned due to a lower number of privately insured patients. Privately insured patients are considered financially attractive as their reimbursement schemes are considered to be more lucrative than statutory health insurance schemes. The concern is that these negatively associated working conditions could influence the next generations of primary care physicians and discourage them to take up a career in rural areas.

### Research question

The aim of this analysis was to explore if working condition factors specifically working time, number of patients treated per week or proportion of insurants of private health insurances differ between practices in rural and urban settings as well as to explore the influences affecting these factors. To answer these questions a secondary analysis of data collected in German primary care physicians within the 2009 physician survey of the Commonwealth Fund conducted by our department was performed.

## Methods

In 2009, the Commonwealth Fund (CWF) conducted the primary care physician survey which is repeated every three years. A questionnaire was sent out to eleven countries during February and July 2009 by Harris Interactive on behalf of the CWF. These countries were: Australia, Canada, Germany, France, Italy, The Netherlands, New Zealand, Norway, Sweden, The United Kingdom and The United States of America.

The sampling and census in Germany was conducted by the Institute for Quality and Efficiency in Health Care (IQWiG) and the department of the authors being the Department of General Practice and Health Services Research of the University Hospital Heidelberg. The authors were therefore authorized to perform the reported exploratory, secondary analysis with the data raised in Germany. A representative random sample of 1500 primary care physicians from all federal states of Germany was chosen based on data of the National Association of Statutory Health Insurance Physicians. Within the German part of the survey all data were pseudonymised. The sample of primary care physicians included general practitioners, general internists and paediatricians. Details of the survey are shown elsewhere [13,14]. Data regarding working hours, numbers of patients in a typical week and the percentage of privately insured patients were retrieved for analysis. Participants working less then 40 hours per week were categorised as part-time working.

### Data analysis

Continuous data were summarized using means and standard deviations. Categorical data were displayed as frequency counts and percentages. Steps of the analysis concerning the workload and the proportion of privately insured patients per practice were performed based on self assessment of participants concerning working in an urban (city or suburb), small city or rural practice. The second step of the analysis involved analysing western versus eastern federal states as eastern federal states are, according to the OECD definition, defined as being almost exclusively rural. Another independent variable was single or group handed practice. Single handed practice in our context means that one physician works alone in his practice whereas a working partnership among more than one physician in a practice is considered a group handed practice. Group comparisons were made by means of Student t test for continuous variables, Chi^2 ^test for categorical variables or ANOVA with Bonferroni correction for post-hoc tests with list wise exclusion of missing data as appropriate. Three linear regression models were performed using the items "working time", "number of patients seen per week" and "proportion of privately insured patients" as dependent variables. The analyses were performed using SPSS version 18.0 (SPSS Inc., Chicago IL, USA). An alpha level of p < 0.05 was used for tests of statistical significance.

## Results

The response rate in Germany was 49% (715 participants). Of the 1500 physicians in the primary sample, 49 addresses were incorrect and therefore the questionnaires were returned unanswered. Of the non participating physicians 123 sent back a postcard with basic sociodemographic data.

### Sample composition

The sample did not differ more than 3% from data of the National Association of Statutory Health Insurance Physicians on primary care physicians in Germany in sex, federal state and specialty [15]. Furthermore there were no differences in sociodemographic data between participants and non participants who returned a postcard instead of the survey. Table 1 shows the sociodemographic characteristics of the study sample.

**Table 1:** Sociodemographic characteristics of the study sample (n = 715)*

	Total number (percent)	City	Small town	Rural area	p-value
Age		n = 232	n = 272	n = 192	0.950

< 35	4 (0.6)	1 (0.4)	2 (0.7)	1 (0.5)	

35-49	271 (38.9)	92 (39.7)	102 (37.5)	77 (40.1)	

50-64	373 (53.6)	126 (54.3)	146 (53.7)	101 (52.6)	

≥ 60	48 (6.9)	13 (5.6)	22 (8.1)	13 (6.8)	

Gender		n = 230	n = 286	n = 193	0.620

Male	444 (64.3)	142 (61.7)	176 (65.7)	126 (65.3)	

Female	247 (35.7)	88 (38.3)	92 (34.3)	67 (34.7)	

*n varies due to missing data

### Working time

Within single handed practices we found a significant difference regarding average working hours with 51.2 hours in urban practices and 55.2 in rural practices (p < 0.05). In group practices there were no significant differences regarding average working hours with 48 hours in urban practices and 51 in rural practices respectively. Numbers of participants working less then 40 hours per week did not differ significantly between urban area (13.4%), small town (12.9%) or rural area (11.4%). On average, physicians worked two hours more in eastern states than those in western federal states; however this difference was also not statistically significant.

### Number of patients seen per week

There was no significant difference between the number of patients seen per week, in urban and rural areas. However, a trend towards more favourable conditions, e.g. seeing fewer patients per week, was found in urban practices. Within one typical week, physicians in single handed rural practices saw about 38 more patients than their urban colleagues and rural group practices were frequented by 21 more patients per week than those located in an urban area. These differences were even more pronounced when comparing western and eastern practices. In single handed eastern practices physicians saw significantly more patients (69 patients) than their western colleagues (p < 0.001). Table 2 shows the working conditions when comparing types of practice and urban or rural practice location defined according to physician self-rating.

**Table 2:** Working conditions comparing type of practice and urban or rural practice location defined according to self-rating (N = 667)

	Urban area (n = 220; Single n = 126; Group n = 94)	Small town (n = 262; Single n = 125; Group n = 137)	Rural area (n = 185; Single (n = 98); Group (n = 87)	p-value
Working time per week Mean (SD)				

Single practice	51.2 (12.0)	51.8(12.4)	55.2 (12.7)	0.042

Group practice	48.3 (12.5)	50.1 (14.6)	50.7 (13.7)	0.469

Patients seen per week Mean (SD)				

**Single practice**	234.8 (121.2)	258.8 (150.5)	272.8 (122.4)	0.094

≤100	18.6%	16.5%	9.2%	0.416
	
101-200	29.5%	26.8%	30.6%	
	
201-300	29.5%	26.0%	30.6%	
	
≥300	22.5%	30.7%	29.6%	

				

**Group practice**	231.8 (133.9)	234.6 (106.4)	253.4 (132.9)	0.445

≤100	16.8%	13.0%	8.7%	0.213
	
101-200	38.9%	34.1%	38.0%	
	
201-300	21.1%	34.8%	27.2%	
	
≥300	23.2%	18.1%	26.1%	

Proportion of private health insures Mean (SD)				

Single practice	14.5 (15.5)	13.2 (17.7)	11.4 (13.2)	0.332

Group practice	13.7 (13.4)	11.4 (8.0)	11.1 (10.6)	0.717

### Proportion of privately insured patients

There was no significant difference between the proportion of privately insured patients, in urban and rural areas. The proportion of privately insured patients did not differ significantly between urban (15%) or rural areas (11%) or between single or group practices in those areas (urban 14%, and rural areas 11%). However when analysing the proportion of privately insured patients between the western and eastern parts of Germany, the percentages differed substantially between western (15%) and eastern (6%) in single handed practices (p < 0.001) and between western (13%) and eastern (4%) in group practices (p < 0.001). Table 3 shows the working conditions in urban or rural practices defined according to OECD.

**Table 3:** Working conditions in urban or rural practices defined according to OECD (N = 684)

	West (n = 569; Single n = 276; Group n = 293)	East (n = 115; Single n = 84; Group n = 31)	p-value
Working time per week Mean (SD)			

Single practice	52.2 (13.0)	54.0 (9.6)	0.251

Group practice	49.6 (13.7)	50.3 (13.8)	0.801

Patients seen per week Mean (SD)			

Single practice	236.0 (129.5)	305.1 (129.7)	< 0.001

Group practice	239.1 (124.2)	270.5 (127.0)	0.183

Proportion of private health insures Mean (SD)			

Single practice	15.2 (16.7)	5.7 (17.7)	< 0.001

Group practice	12.8 (10.7)	4.4 (2.7)	< 0.001

### Influencing Factors

Table 4 shows the factors influencing working time, proportion of privately insured patients and number of patients seen per week analysed by a regression analysis with the dependent variables working time, proportion of privately insured patients and number of patients per week. We identified that gender, number of patients per week and type of practice (single/group handed) were significantly associated with the amount of working time/working hours per week. These 3 factors explained more than 20% (r^2^~0.20) of the variance of the dependent variable working time. Additionally we identified that single or group practice, age, patients per week and localisation in either a western or eastern federal state were significantly associated with the proportion of privately insured patients. These 4 variables explained more than 10% (r^2^~0.10) of the variance of the variable. For numbers of patients seen per week, we identified that working time, localisation in either a western or eastern federal state and proportion of privately insured patients were significantly associated with the number of patients seen per week. These 3 variables explained almost 10% (r^2^~0.096) of the variance of the variable.

**Table 4:** Influences on working time, proportion of privately insured patients and patients seen per week

	Working time		Number of private insured patients		Number patients seen per week	
	**ß (p-value)**	**t**	**ß (p-value)**	**t**	**ß (p-value)**	**t**

Gender	-.167 (**< .001)**	-4.679	.035 (.363)	-.911	-.047 (.195)	-1.298

Age	-.034 (.339)	-.957	-.090 (**< .05)**	-2.413	-.025 (.473)	-0.717

East/West	-.021 (.562)	-.581	-.218 (**< .001)**	-5.711	.111 **(< .01)**	3.065

single/group practice	-.084 (**< .05)**	-2.328	-.106 (<**.01)**	-1.160	-.005 (.881)	-.150

localisation of practice (urban/rural)	.059 (.096)	1.668	-.043 (.246)	-1.160	.035 (.311)	1.014

Mean patients per week	.385 (**< .001)**	10.527	-.198 (**< .001)**	-4.811	__	**__**

Proportion of private insured patients	.005 (.884)	.146	__	__	-.200 **(< .001)**	-4.811

Working time per week	__	__	.006 (.884)	.146	.377 **(< .001)**	10.527

**Pseudo R^2^**		**0.202**		**0.108**		**0.219**

**F (p-value)**		**24.797 (< .001)**		**12.378 (< .001)**		**27.407 (< .001)**

(results of linear regression analysis, under specification of standardized beta coefficient, α = 5%)

## Discussion

Primary care physicians working in single practices in rural areas do work significantly more hours than their urban colleagues. However working hours differ far less between urban and rural areas than GP trainees perceive to be the case. The difference is even less when group practices are compared.

When applying the self-rating approach regarding rurality, working hours in single handed practices differ significantly, however not the number of patients or the proportion of privately insured patients. In contrast, when applying the OECD definition for population density, significant differences regarding the number of patients per week and the proportion of privately insured patients have been identified. However, we are not able to assess to what extent this finding may be influenced by the former East Germany socialistic history. This history may have affected the proportion of privately insured patients, as there was no private insurance system in the former German Democratic Republic. Why GP age is associated with the proportion of privately insured patients should be a matter of future research. Average working hours of physicians working in a hospital in Germany is about 55 hours, which is similar to the working hours we found for single handed practices in rural areas [16]. Our results differ from those of a study by Kroneman et al. who reports on an average of 60 working hours in Germany in the year 2000. Unfortunately within this study working time between urban and rural areas was not reported separately [17]. A Canadian study based on data from the year 1998, showed a difference of eight hours between working time in urban and rural areas [18]. These differences might be due to changes in practice management during the last 10 years. Attitudes towards work-life-balance also seem to have changed in favour towards leisure time or family obligations which might have an impact on working time [19].

One reason for misjudging working conditions in primary care in Germany might be due to the fact that many GP trainees work in a single handed practice during their vocational training period and therefore experience a higher burden of work with more working hours than colleagues in group practices [20]. Additionally, we know that many GP trainers complain about the current circumstances in the health care system and the high work load which has a high impact on job satisfaction [14,21]. Therefore, they may have an influence on the perceptions of students and trainees resulting in a substantial negative impact on career choice towards primary care in rural areas [22].

The number of patients seen per week is the highest in Germany, compared to the eleven participating countries [14]. This high workload might also influence physicians' satisfaction. Although we identified that working time, localisation in either a western or eastern federal state and the proportion of privately insured patients are significantly associated with the number of patients seen per week, the highest impact on the number of patients seen per week is most likely the reimbursement system in Germany [23]. The reimbursement system works in a way that more patient contacts are rewarded by higher income.

Unlike data reported in research from Beardow, our data shows that the proportion of women working in rural or urban areas are quite similar ranging between 34% to 38% [24]. This might be due to different characteristic of rurality in Germany compared with rurality in countries such as Australia and Canada. Although in our data gender is associated with working hours we have to question whether this is a specific gender effect or a reference to necessary changes in society. In Germany there is an ongoing discussion about impact and consequences of the *feminisation *within medicine. Data of 2007 show that 27% of female physicians and 7% of male physicians work part-time [25]. The estimation that three female physicians are necessary to equal the lifetime workforce of two male physicians has not yet been critically evaluated [26]. Work-life-balance issues leading to part time working might be an issue of changes in society rather than a gender specific one [19,27]. Since employees have recently focused on improving working conditions for physicians in Germany, future gender specific differences regarding this aspect might be less relevant.

The relevance of residents having rural area experience when choosing a career in a rural area is widely known [28]. As a consequence, strategies to correct misdistribution by admitting students from rural backgrounds preferentially into university and offering clinical training in rural settings have been implemented [29]. From our analysis we would conclude that early direct contact with rural areas during medicine school could reduce negative perceptions towards working in a rural area.

As a next step a questionnaire addressing factors important for medical care in rural areas will be designed for Germany to enable a more precise definition of rurality in future studies [8,30-32].

### Strengths and Limitations

The strength of this study is the representative random sample of 1500 primary care physicians that was chosen on behalf of the data of the National Association of Statutory Health Insurance Physicians from all federal states in Germany. As participants are comparable to the overall physician population in Germany, systematic bias due to demographic reasons is less likely. There were no sociodemographic differences between participants and non participants returning a postcard instead of the survey. However there are methodological limitations including selection and recall bias for answers regarding working time and patients seen per week. A further limitation is that there was no explicit item in the questionnaire asking whether the physician works full-time or part-time. Future studies should include this question. However as described within our results section the numbers of participants working less than 40 hours per week did not differ significantly between urban area, small town or rural area. Furthermore both definitions of rurality used have limitations. The OECD definition can not distinguish whether or not a participating practice in East Germany is located in a rural area. By self-rating of participating GPs on the other hand, perceptions towards rurality can differ according to individual biography, experiences with other healthcare systems and geography. There are also important differences in terms of the number of GPs in the sample located in the West compared with the East, as West and East are defined by history and the eastern part is smaller. The balance between single and group practices in East and West is also partly influenced by the former history as a single health care system for both regions only exists since 1990.

## Conclusion

Although single practice GPs in rural areas do work significantly more hours per week, often stated prejudices towards rurality concerning working hours, number of patients and the proportion of privately insured patients could not be confirmed within this survey. Rather, it must be concluded that hindering factors associated with working in rural areas are much more complex and need to be evaluated in depths within future research.

Future health care concepts in Germany should include more group practices as working hours between urban and rural areas (independent of the definition) are much more comparable than in single handed practices.

The OECD definition of rurality cannot easily be used in health care research as it has too many limitations. An instrument, measuring from a health care point of view, what rurality means, needs to be developed for each country. With the results of such an instrument, strategies facing physician shortage in rural areas might be tailored differently. Therefore strategies could be different in western or eastern federal states. Additional, effective strategies facing the shortage of primary care physicians in rural areas must counter subjective aspects by permitting students and residents to get hands on experience in rural areas.

## Competing interests

The authors declare that they have no competing interests.

## Authors' contributions

JS drafted the manuscript and analysed the data together with AM. SJ, JSz and AM conducted the study and made contributions to the manuscript. All authors read and approved the final manuscript.

## Pre-publication history

The pre-publication history for this paper can be accessed here:

http://www.biomedcentral.com/1471-2296/12/112/prepub
